# Reconstruction of Soft Tissue Defects in the Hand with a Free Anterolateral Thigh Deep Fascia Flap

**DOI:** 10.1111/os.12948

**Published:** 2021-03-05

**Authors:** Li Wang, Huiren Liu, Tiepeng Ma, Xueqiang Wu, Liu Zhang

**Affiliations:** ^1^ Department of Orthopedic Surgery Hebei Medical University Shijiazhuang China; ^2^ Department of Hand Surgery The Second Hospital of Tangshan Tangshan China; ^3^ Department of Orthopedic Surgery Emergency General Hospital Beijing China

**Keywords:** Anterolateral thigh flap, Deep fascia flap, Hand, Reconstruction, Soft tissue defect

## Abstract

**Objective:**

To report our experience in the reconstruction of soft tissue defects in the hand with a free anterolateral thigh deep fascia flap and describe the clinical outcomes.

**Methods:**

This study was a retrospective trial. From November 2016 to January 2020, six patients (four men and two women) with soft tissue defects in the hand were included in this study. The average age of the patients was 33.7 ± 12.7 years (range, 20 to 50 years). All patients underwent reconstructions with free anterolateral thigh deep fascia flaps. Relevant clinical characteristics were recorded prior to surgery. The size and thickness of the deep fascia flap and the thickness of the skin were measured intraoperatively. The survival of the flaps and skin grafts and the occurrence of infection were recorded after the operation. At follow‐up, donor site complications and postoperative effects were evaluated according to the outcome satisfaction scale. The pain in the injured hand was assessed using the visual analog scale.

**Results:**

The average body mass index (BMI) was 26.6 ± 1.7 kg/m^2^ (range, 23.9 to 28.7 kg/m^2^). The defect sizes ranged from 5 cm × 5 cm to 13 cm × 8 cm (average, 53.1 ± 27.9 cm^2^). The six anterolateral thigh deep fascia flaps ranged from 7 cm × 6 cm to 14 cm × 9 cm in size (average, 71.8 ± 29.1 cm^2^). The thicknesses of skin ranged from 25 mm to 40 mm (average, 32.5 ± 4.8 mm), and the thicknesses of the deep fascia flaps ranged from 2 mm to 3 mm (average, 2.5 ± 0.5 mm). After the operation, the blood supply of the deep fascia flap was normal in all cases. The second‐stage skin grafts of most patients survived completely. The skin graft in one case was partially necrotic and healed after a dressing change. No infection occurred. At follow‐up (average, 16.3 ± 6.9 months), there was only a linear scar and no loss of sensation at the donor site of each patient. According to the outcome satisfaction scale, the outcome satisfaction score ranged from 6 to 8 (average, 7.2 ± 0.9), all of which were satisfactory. Apart from one patient who reported mild pain, all the other patients reported no pain. Three typical cases are presented in this article.

**Conclusions:**

The free anterolateral thigh deep fascia flap, which is suitable for reconstruction of soft tissue defects in the hand, can provide very good outcomes both functionally and aesthetically.

## Graphical abstract

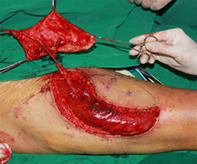

We present a newly free anterolateral thigh deep fascia flap. The flap, which can avoid the bulky appearance of the traditional anterolateral thigh flap and involves fewer complications at the donor site, is suitable for the reconstruction of soft tissue defects in the hand, especially irregular defects in obese and/or female patients. It can provide good outcomes both functionally and aesthetically.

## Introduction

Disability of the hand affects not only people's life and labor but also their appearance and social interactions. Effective and aesthetic coverage of soft tissue defects is key in the treatment of hand injuries and is directly related to the recovery of hand function. Nevertheless, reconstruction of soft tissue defects in the hand remains a great challenge for orthopedic and plastic surgeons, especially when tendons and bone are exposed. The ideal reconstructive tissue mainly needs to have the following characteristics^1^: similar color and texture to the recipient area, relative tightness, thinness and pliability of the flap for molding the hand contours, cause no hindrance to the range of hand motion, enhance both functional and esthetic outcomes, minimal donor‐site morbidity, and no change in intraoperative position. In the past, retrograde radial fasciocutaneous forearm flaps, posterior interosseous flaps, lateral arm flaps and other flaps were used to reconstruct defects^1^. However, these flaps are limited by the relatively bulky and short pedicle, as it is dangerous for the blood supply to directly close the pedicle and difficult to extend to the distal hand after rotation. Elevation of these flaps may cause functional deficits and potential morbidity of the donor site, and poor aesthetic outcomes may be observed after skin graft of the donor site.

With the development of microsurgery, the application of free flaps in hand defects is gradually increasing, e.g., the paraumbilical perforator flap, latissimus dorsi flap, anterolateral thigh (ALT) flap, and tensor fasciae latae flap^2^. The ALT flap, which was first introduced by Song *et al*. in 1984^3^, is a fasciocutaneous flap based on septocutaneous or musculocutaneous perforators supplied by the lateral femoral circumflex system; it was further developed and popularized for clinical application by Koshima *et al*.^4^, ^5^, ^6^ and Wei *et al*.^7^. With a great deal of versatility and reliability, the ALT flap has become the standard flap for a variety of soft tissue reconstructive operations of the extremities and trunk. Some surgeons applied the ALT flap to reconstruct soft tissue defects in the hand and achieved encouraging results^8^, ^9^, ^10^. The ALT flap is pliable and soft and can be harvested with or without fascia and fat. It has many advantages, including a long pedicle with a suitable vessel diameter, a relatively consistent vascular anatomy, a variety of widths and depths of tissue for coverage, adaptability as a sensate or flow‐through flap, and acceptable donor‐site morbidity. One drawback of the ALT flap, especially in obese and/or female patients, is that it is too bulky, making the flap inset difficult and possibly compromising flap circulation. Bulky flaps cause aesthetically unsatisfactory outcomes and poor functioning reconstructions in the hand. Therefore, secondary defatting of the flap is often required to improve the appearance and function of the hand. Despite thinning, the hairy appearance of the ALT flap may induce poor long‐term outcomes.

With the significant improvement in the flap survival rate, orthopedic surgeons shifted their interest in flaps from survival to aesthetic and functional outcomes, which is achieved by thinning the flap^1^, ^11^. Flap thinning refers to the removal of excess adipose tissue from the flap while preserving adequate circulation. The thinning techniques could be classified into three main approaches^12^: defatting after flap elevation (i.e. the removal of specific fat components after conventional flap elevation), thin elevation with modification of the plane (i.e. the flap elevation relying on superficial fascia) and defatting after thin elevation (combined method). Although thin flaps are useful for resurfacing the hand, only partial or marginal defatting is mostly used^13^, ^14^, ^15^, ^16^, ^17^. An excessive defatting procedure may cause flap necrosis because of the destruction of perforators. The thin flap generally tends to be hyperemic relative to a traditional flap, and hematoma may compromise circulation. This means that vascular‐related complications are more common in thin flaps^2^, ^18^, ^19^, ^20^. For this reason, some authors do not recommend one‐stage thinning techniques but suggest secondary liposuction^19^, ^20^, ^21^. Similar to pedicled flaps and traditional ALT flaps^22^, donor site complications of thin ALT flaps sometimes occur and mainly include some loss of sensation at the ALT aspect and limited range of motion of the hip and knee joint because of adhesions between the meshed skin graft and the underlying fascia. In addition, wider flaps may increase donor site morbidity.

At present, the method of reconstruction for soft tissue defects in the hand is still controversial because of associated problems, such as thinning the flap, ensuring the blood supply and minimizing donor‐site morbidity. We retrospectively analyzed six cases of reconstructing the soft tissue defect in the hand with a free ALT deep fascia flap from November 2016 to January 2020. After the second‐stage skin grafting, the appearance and function of the hands were good, and there was little damage to the donor site. As far as we know, this method is rarely reported. Accordingly, the purposes of this study were: (i) to evaluate the clinical effect of this method; (ii) to analyze the advantages of this method and identify its limitations; and (iii) to point out the indications for this method and put forward directions for further development.

## Patients and Methods

The ethical committee of our hospital approved the study protocol. Written informed consent was obtained from all patients. All investigations were conducted according to the principles expressed in the Declaration of Helsinki.

### Inclusion and Exclusion Criteria

Inclusion criteria were as follows: (i) soft tissue defects in the hand with exposure of tendon or bone; (ii) patients treated with a free ALT deep facia flap; (iii) comparison of preoperative and postoperative status; (iv) evaluation of surgical procedure and postoperative status; and (v) retrospective study. Exclusion criteria were patients with: (i) severe brain or organ damage; (ii) severe damage on the same limb (affecting the blood supply); (iii) psychiatric disorders, coagulation disorders, diabetes or smoking history; and (iv) lost clinical data.

### General Information

From November 2016 to January 2020, six patients (4 men and 2 women) with soft tissue defects in the hand were treated with the free deep fascia of an ALT flap. The average age of the patients was 33.7 ± 12.7 years (range, 20 to 50 years), and the average body mass index (BMI) was 26.6 ± 1.7 kg/m^2^ (range, 23.9 to 28.7 kg/m^2^). The cause of the soft tissue defects was industrial machinery injuries. All soft tissue defects were complicated by tendon and/or bone exposure requiring coverage reconstruction. The defect sizes ranged from 5 cm × 5 cm to 13 cm × 8 cm *(average, 53.1 ± 27.9 cm^**2**^). There was no underlying disease in the 6 patients**.***

### Flap Design

**Fig. 1:**
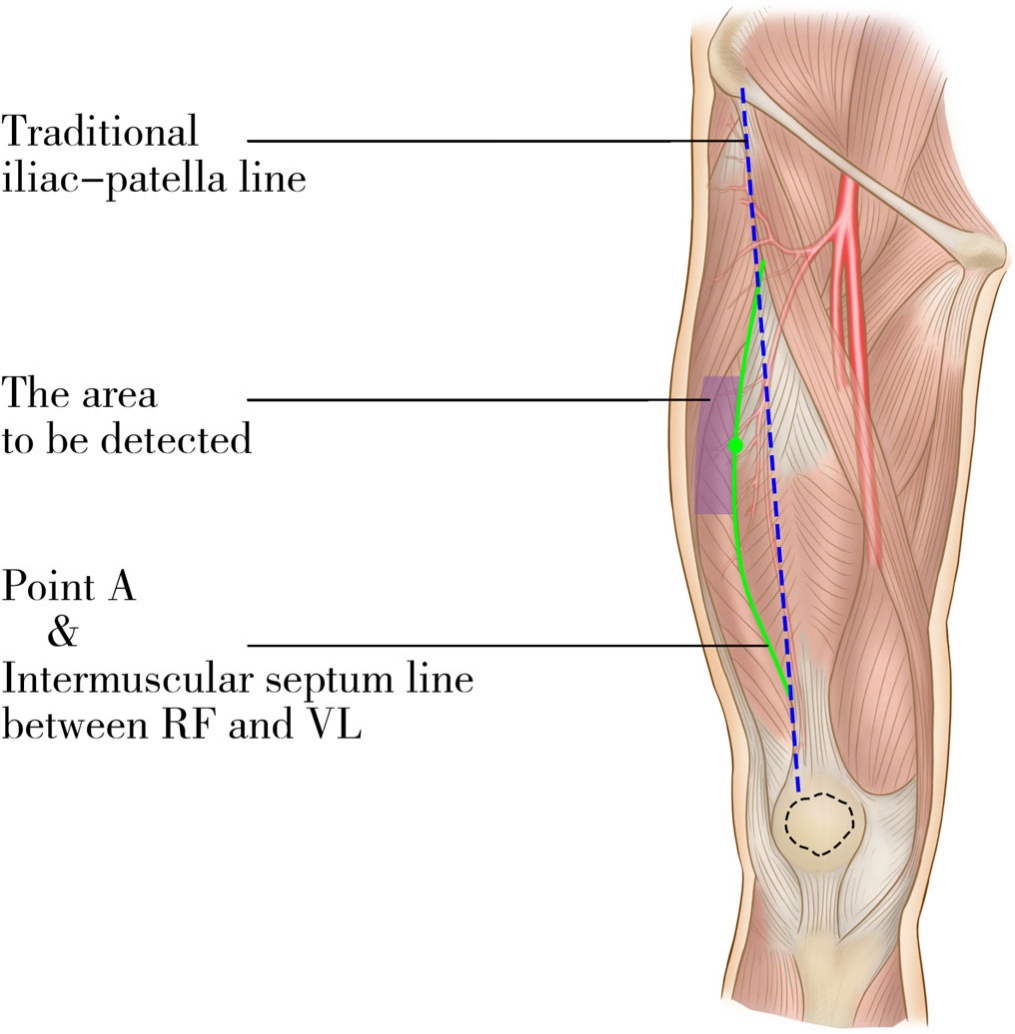
Blue dotted line: the traditional iliac‐patella line. Green line and point: the intermuscular septum line between rectus femoris (RF) and vastus lateralis (VL) & point A. Purple area: the area for Doppler detection preoperatively.

Before anesthesia, the patient in the supine position initiatively raised the straight lower limb at the donor site. The intermuscular septum line between the rectus femoris (RF) and vastus lateralis (VL) was identified by palpation, and the line was designated “line a”. The intersection of “line a” and the middle thigh was set as “point A”. Within the lateral 3 cm of “line a” and within the upper and lower 5 cm of “point A”, a hand‐held Doppler was used to detect the exit points of cutaneous perforators. (Fig. 1) The characteristics of the exit point are that the vascular pulsating sound is punctate and the sound is weakened after being pressured slightly. The number of exit points is generally 2–3. According to the location of the exit point, the flap was designed preliminarily to make the point located in the center of the upper one‐third flap. The size of the deep fascia flap was 1–2 cm larger than that of the defect. The flap was redesigned after the exact location of the exit point was determined intraoperatively. Preoperative hand‐held Doppler examination was not always accurate^23^.

### Surgical Technique

#### Anesthesia and Position

The patient was placed in a supine position under general anesthesia. Two teams worked simultaneously: the hand team prepared the recipient site, and the flap team harvested the flap.

#### Approach and Flap‐Harvesting (The FLap Team)

*To Explore the Perforators and their Main Vascular Pedicle*. According to the design, the lateral margin and part of the upper margin of the flap were incised down through the skin and subcutaneous tissue to the deep fascia over the VL. The flap was turned inward in the subfascial plane for the identification of perforators. The strongest perforator was selected, and the position of the flap was adjusted according to this position. Alternatively, according to the needs of the defects, multiple perforators were selected to design the multilobed deep fascia flap. At the upper margin of the flap, the proper incision was extended toward the beginning of the femoral artery, deep to the subfascial plane. The descending branch of the lateral circumflex femoral artery was explored between the RF and VL. The tendency of the perforator toward the main vascular pedicle was identified.

*To Dissect the Perforators*. At the exit point of the perforator, the muscle fibers were dissected along the course of the penetrating branch down to the main vascular pedicle. The VL fibers located on the superficial layer of the perforator were cut off through the bipolar electrocoagulation technique. All visible caliber vessels were ligated to ensure a bloodless operation field. The motor nerves in VL should be preserved. When the perforator and its main vascular pedicle had been completely exposed, they were separated from the muscles below them with no fibers taken.

*To Harvest the Deep Fascia ALT Flap and Close the Donor Site*. Then, the deep fascial part of the ALT flap was separated from the subcutaneous tissue, and a thin layer of adipose tissue was preserved on its surface. With the other three margins of the deep fascia flap cut off, the deep fascia flap was elevated completely while the perforator was preserved. According to the requirement of the pedicle length, the main vascular pedicle was cut and ligated at the proper location after the circulation was confirmed. The skin incision in the donor area could always be closed directly.

#### Reconstruction (The Hand Team)

After debridement, the area and shape of the defect were measured for the flap design. After receiving the free deep fascia flap, the deep fascia flap was trimmed to suit the defect. Then, the hand defect was covered with a deep fascia flap and sutured. Through the subcutaneous tunnel, the vascular pedicle was guided to the normal tissue and was anastomosed with two superficial veins and the radial or ulnar artery.

### Procedure after Flap Operation

The dressing change was performed once a day, and the hand was protected by forearm plaster. Two weeks after the flap operation, the deep fascia flap obtained stable circulation, and skin grafting was performed. The adipose tissue on the surface of the deep fascia flap should be properly cut off to improve the survival rate of skin grafting.

### Data Collection and Evaluation of Outcomes

The deep fascia flap size, the number of detected perforators, the thickness of skin, and the thickness of deep fascia flap were measured intraoperatively, and the thinned thickness was obtained by the thickness of skin minus the thickness of deep fascia flap. The survival of the flaps and skin grafts and the occurrence of infection were recorded after the operation.

#### Outcome Satisfaction Scale^24^

At follow‐up (average, 16.3 ± 6.9 months; range, 6 to 24 months), donor site complications and postoperative effects were evaluated according to the outcome satisfaction scale developed by Zhang *et al*.^24^ The score includes five aspects, namely, wound healing, flap shape, flap sensation, flap temperature and donor scarring. Among them, the excellent score is 2 points, the good score is 1 point, the fair score is 0 points, and the poor score is −1 point. Add up the scores in each aspect to calculate the total score. A total score of 5–10 indicates satisfaction; 0–4 indicates average; −1–5 indicates dissatisfaction.

#### Visual Analog Scale (VAS)

A 10‐cm‐line visual analog scale (VAS) was used to evaluate pain sensations of the injured hand, which was categorized into painless (0 cm), mild (1–3 cm), moderate (4–6 cm), and severe (7–10 cm).

## Results

### General Results

The time interval between the injury and treatment ranged from 7 days to 21 days (average, 14.2 ± 4.7 days). The six ALT deep fascia flaps ranged from 7 cm × 6 cm to 14 cm × 9 cm in size (average, 71.8 ± 29.1 cm^2^). A total of 13 perforators were detected (average, 2.2 ± 0.7), all of which originated from the descending branch of the lateral circumflex femoral artery, including 11 musculocutaneous perforators (84.6%) and 2 septocutaneous perforators (15.4%). The thicknesses of skin were measured in the surgical incision, which ranged from 25 mm to 40 mm (average, 32.5 ± 4.8 mm). After the deep fascia flaps were harvested, their thicknesses were measured, ranging from 2 mm to 3 mm (average, 2.5 ± 0.5 mm). Thus, the average thinned thickness was 30 ± 4.5 mm, and the range was 23–37 mm. The donor sites at the thigh of all patients were directly closed. After the operation, the blood supply of the deep fascia flap was normal in all cases, and venous congestion was not observed. The second‐stage skin grafts of most patients survived completely. The skin graft in one case was partially necrotic and healed after dressing change. No infection occurred.

### Outcome Satisfaction Scale

During the follow‐up period (average, 16.3 ± 6.9 months), the flaps were not bulky and presented with a normal texture and no ulcers. Each patient's hand recovered part of the function corresponding to his or her injury. There was only a linear scar and no loss of sensation at the donor site thigh of each patient. All the patients retained normal quadriceps muscle strength and the range of motion of their hip and knee joints. Based on the outcome satisfaction scale developed by Zhang *et al*.^24^, the outcome satisfaction score ranged from 6 to 8 (7.2 ± 0.9), all of which were satisfaction.

### Visual Analog Scale (VAS)

According to the VAS, with the exception of one patient (Patient 5) who reported mild pain (2 cm) at the injured hand, all the other patients reported no pain (0 cm).

### Illustrative Case

#### Case 1

**Fig. 2:**
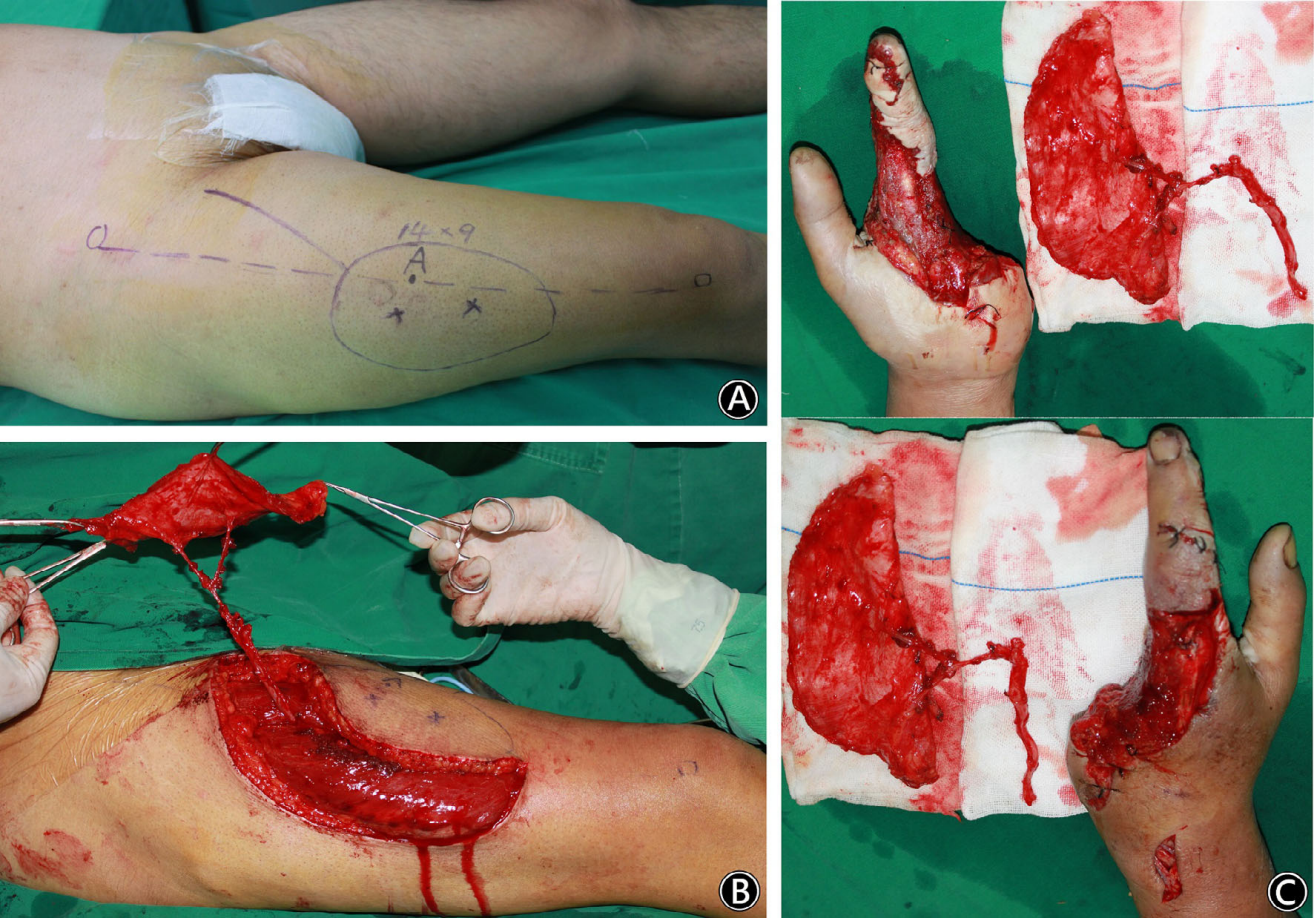
A 50‐year‐old man injured his left hand by a rail wheel at work. (Case 1). (A) Flap design to show two perforators outside the intermuscular septum line. (B) Intraoperative elevation of the anterolateral thigh (ALT) deep fascia flap. (C) The soft tissue defect in the left hand & the intraoperative view after harvest of the free ALT deep fascia flap.

**Fig. 3:**
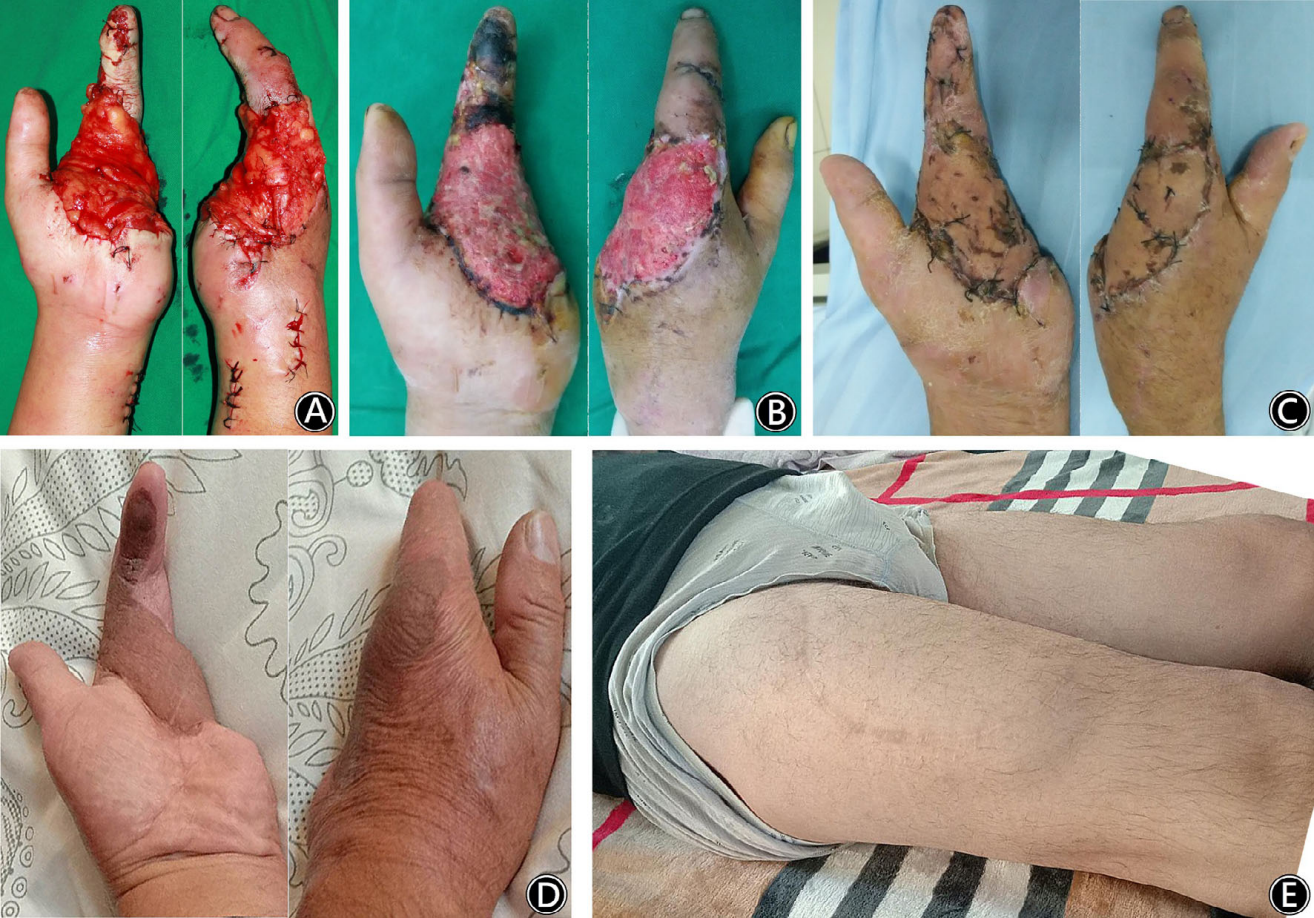
A 50‐year‐old man injured his left hand by a rail wheel at work. (Case 1). (A) The intraoperative view after transfer of the free ALT deep fascia flap. (B) Two weeks after the flap operation, the deep fascia flap obtained stable circulation. (C) The split skin graft survived completely. (D) At follow‐up, the flap had good circulation and presented a nonbulky appearance. (E) At follow‐up, there were no complications at the donor site.

A 50‐year‐old man injured his left hand by a rail wheel at work. The limb far from the third to fifth metacarpal bone was seriously damaged, and the skin on the index finger was defective. One week after emergency debridement, soft tissue reconstruction of the left hand was performed. The left hand defect was 13 cm × 8 cm in size, with tendon and bone exposed. A free ALT deep fascia flap with a size of 14 cm × 9 cm was harvested from the right thigh to cover the left hand defect. Two weeks after the flap operation, the deep fascia flap obtained stable circulation, and a split skin graft was performed. At the follow‐up 2 years after the operation, the flap had good circulation and presented a nonbulky appearance, and the partial function of the left hand recovered. There were no complications at the donor site of the right thigh. (Figs 2, and 3).

#### Case 2

**Fig. 4:**
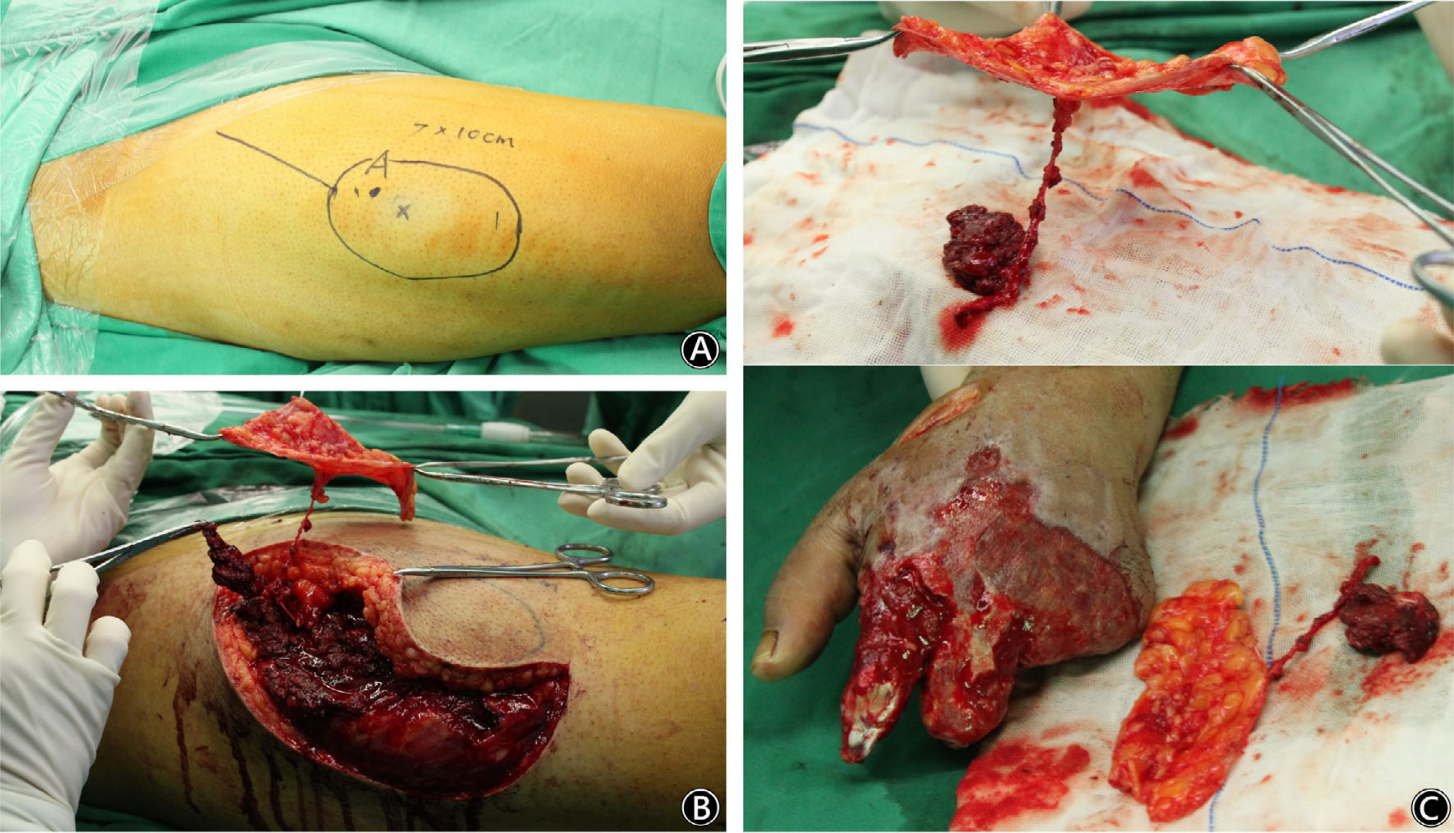
A 21‐year‐old man injured his left hand with a hot roller at work. (Case 2). (A) The flap design. (B) Intraoperative elevation of the ALT deep fascia flap. (C) The soft tissue defect in the left hand & the intraoperative view after harvest of the free ALT deep fascia flap.

**Fig. 5:**
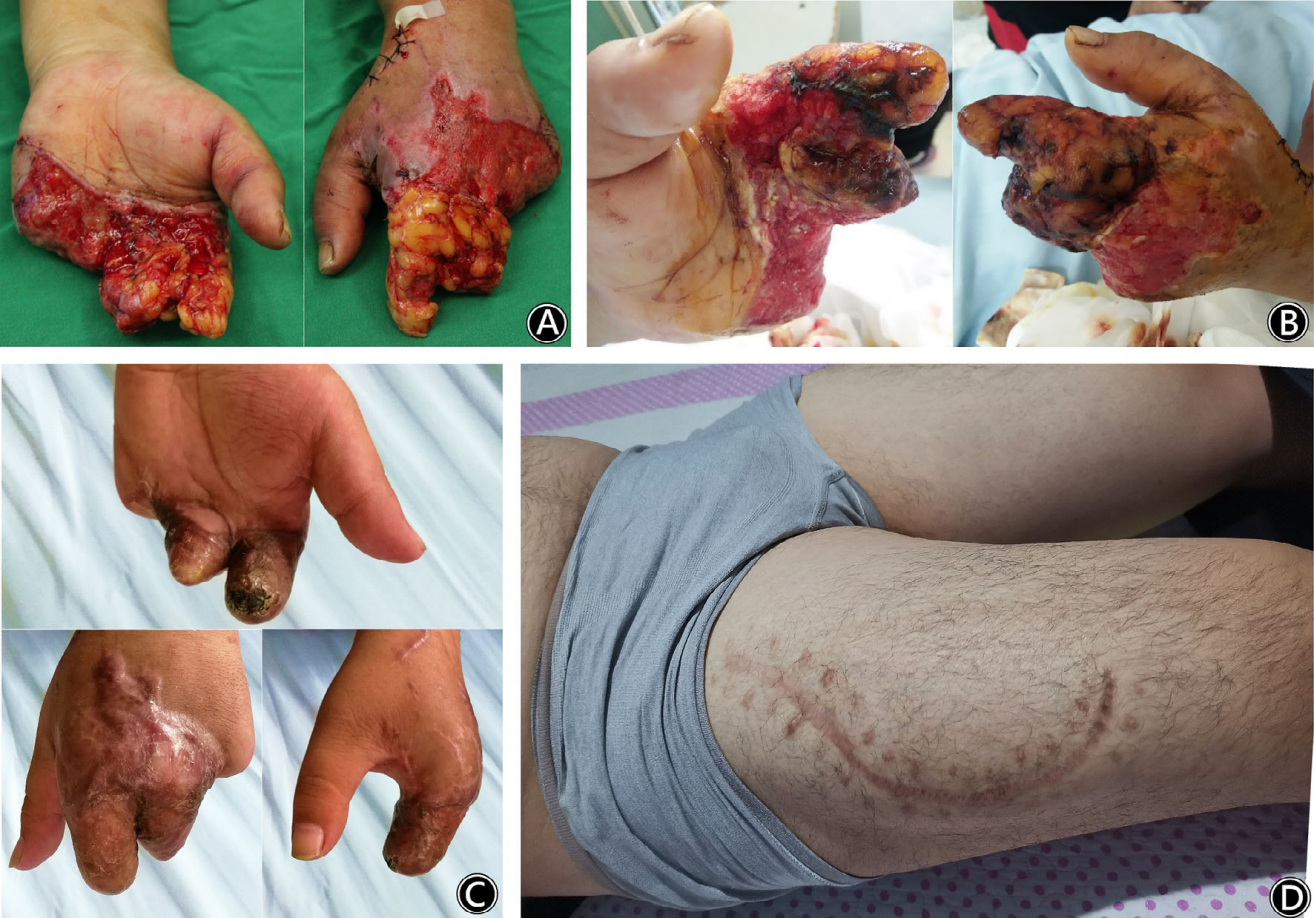
A 21‐year‐old man injured his left hand with a hot roller at work. (Case 2). (A) The intraoperative view after transfer of the free ALT deep fascia flap. (B) Two weeks after the flap operation, the deep fascia flap obtained stable circulation. (C) At follow‐up, the flap had good circulation and presented a nonbulky appearance. (D) At follow‐up, there were no complications at the donor site.

A 21‐year‐old man injured his left hand with a hot roller at work. The ring and little fingers and the distal end of the index and middle fingers were seriously damaged. Two weeks after emergency debridement, soft tissue reconstruction of the left hand was performed. There were many defects in the left hand, including defects in the palm and back and annular defects in the index and middle fingers. The total defect was 9 cm × 6 cm in size, with tendon and bone exposed. A free ALT deep fascia flap with a size of 10 cm × 7 cm was harvested from the right thigh and was tailored to a proper shape for coverage of the left hand defect. Two weeks after the flap operation, the deep fascia flap obtained stable circulation, and a split skin graft was performed. At the follow‐up 1.5 years after the operation, the flap had good circulation and presented a nonbulky appearance, and partial function of the left hand was recovered. There were no complications at the donor site of the right thigh. (Figs 4, and 5).

#### Case 3

**Fig. 6:**
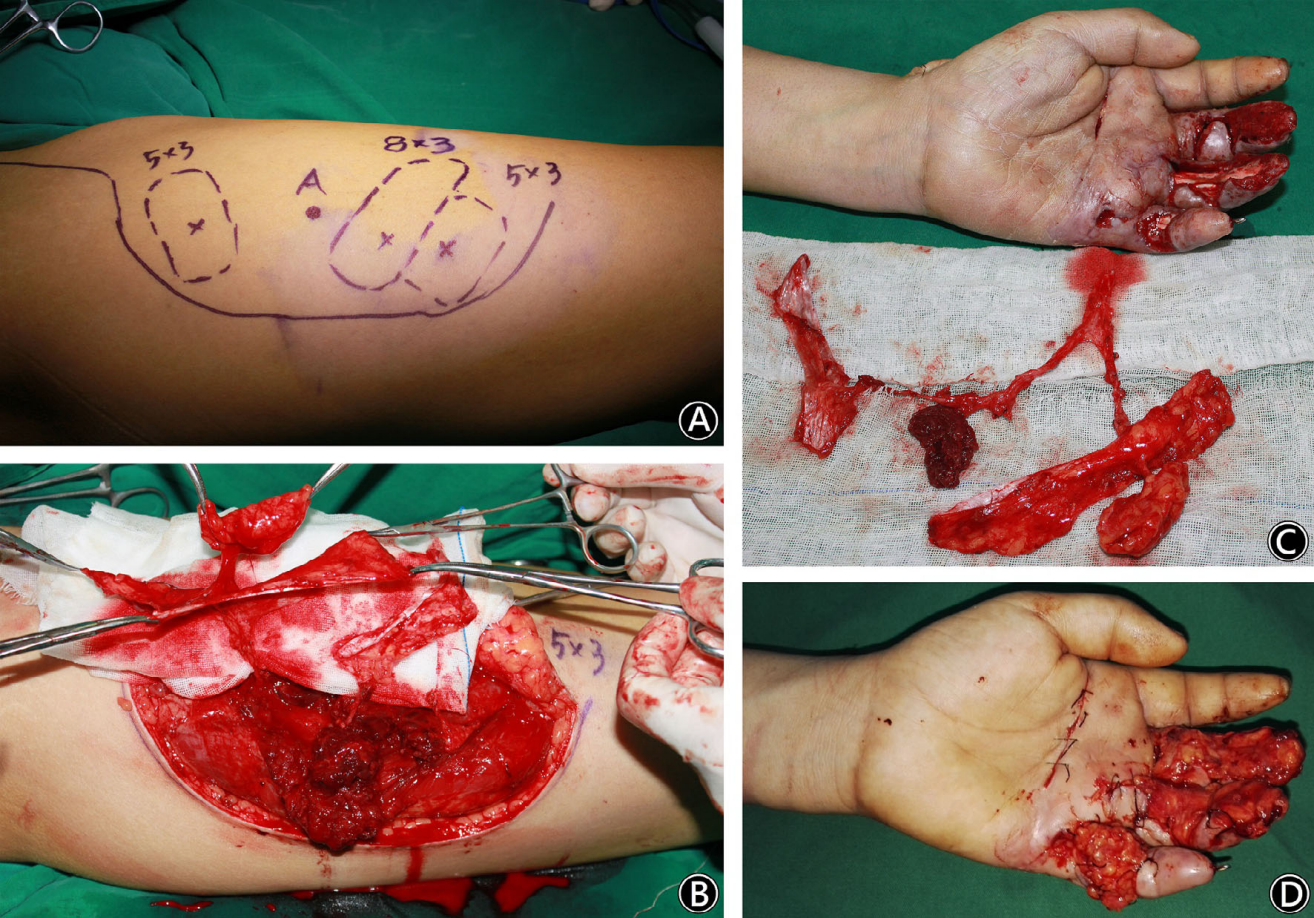
A 24‐year‐old man crushed his 3–5 fingers of the left hand with a machine at work. (Case 3). (A) Operative incision and design of the multilobed ALT deep fascia flap. (B) Intraoperative elevation of the multilobed ALT deep fascia flap. (C) The soft tissue defect in the left hand & the intraoperative view after harvest of the free multilobed ALT deep fascia flap. (D) The intraoperative view after transfer of the free multilobed ALT deep fascia flap.

**Fig. 7:**
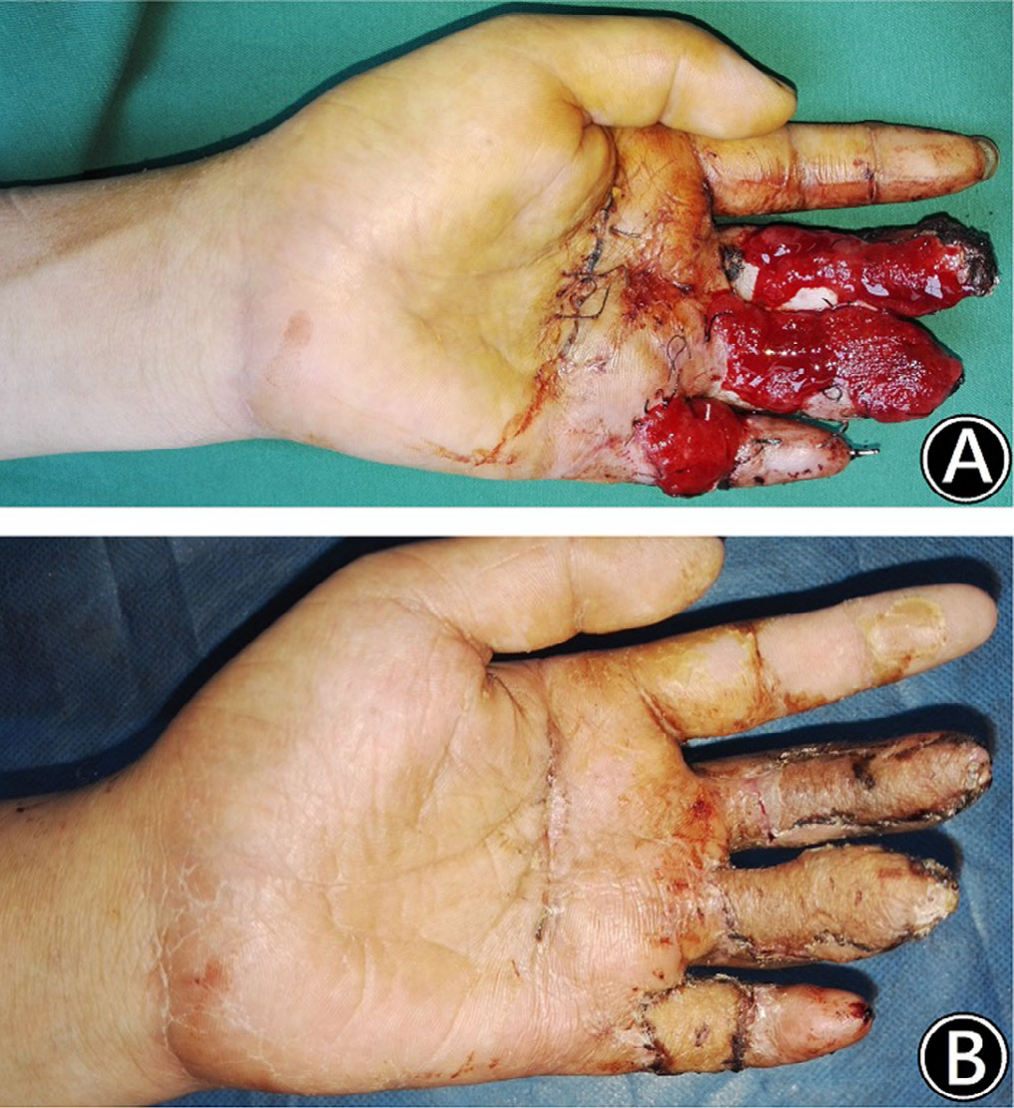
A 24‐year‐old man crushed his 3–5 fingers of the left hand with a machine at work. (Case 3). (A) Two weeks after the flap operation, the deep fascia flap obtained stable circulation. (B) The split skin graft survived completely, and the flap presented a nonbulky appearance.

A 24‐year‐old man crushed his 3–5 fingers of the left hand with a machine at work. The skins of 3–5 fingers were defective. Three weeks after emergency debridement, soft tissue reconstruction was performed. There were soft tissue defects with tendons exposed in the 3–5 fingers of the left hand, and the defects were 4.5 cm × 2 cm, 5.5 cm × 2 cm, and 3.5 cm × 2.5 cm, respectively. Using two perforators, a free multilobed ALT flap was harvested from the right thigh to cover the finger defects. Two deep fascial lobes were 5 cm × 3 cm and 8 cm × 3 cm in size, and one subcutaneous lobe was 5 cm × 3 cm in size. Two weeks after the flap operation, the multilobed flap obtained stable circulation, and a split skin graft was performed. At the follow‐up 6 months after the operation, the flap had good circulation and presented a nonbulky appearance, and there were no complications at the donor site of the right thigh. (Figs 6, and 7).

## Discussion

### Mechanism of this Technique and Summary of the Major Results of the Study

**Fig. 8:**
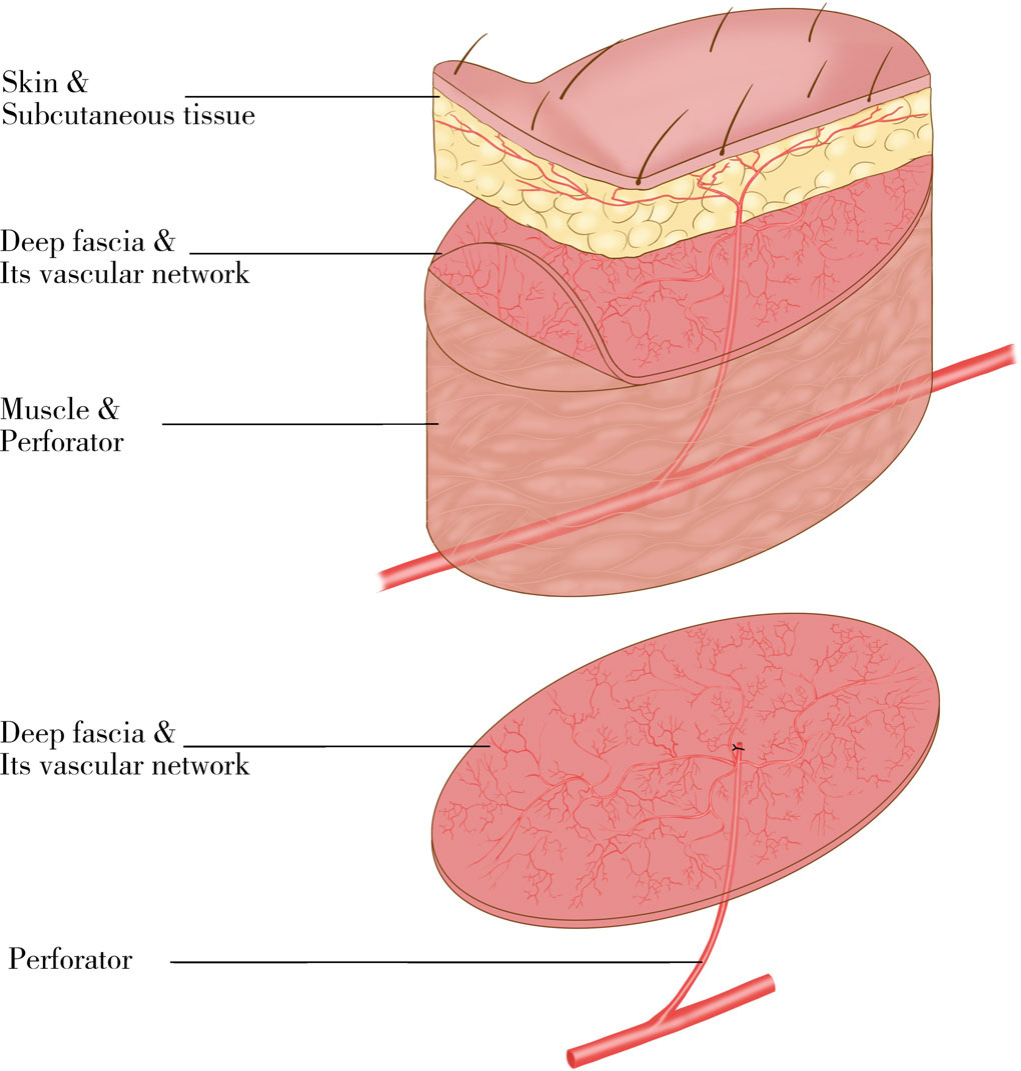
(up) Schematic diagram of the blood supply of the skin. (down) Schematic diagram of the blood supply of the deep fascia flap.

The rich vascular network in the deep fascia has been emphasized by orthopedic and plastic surgeons. Of all the constituents of a fasciocutaneous flap (i.e. epidermis, dermis, subcutaneous tissue, and deep fascia), the deep fascia has the most significant vascular network^25^. Musculocutaneous and septocutaneous perforators first form the vascular plexus at the level of the deep fascia as they perforate it on their way to the superficial layers of anastomotic channels. (Fig. 8) Over the years, surgeons have made full use of this knowledge to design flaps of various components according to the functional and aesthetic requirements of the recipient areas^26^, ^27^, ^28^. On the basis of the vascular network in the deep fascia, we reconstructed six cases of soft tissue defects in hands with free ALT deep fascia flaps. At follow‐up, there was only a linear scar and no loss of sensation at the donor site of each patient. According to the outcome satisfaction scale and the VAS, all of patients were satisfactory, and 83.3% of patients reported no pain.

### Advantages of the ALT Deep Fascia Flap

Despite the advantages of the ALT flap mentioned earlier, the ALT deep fascia flap also has the following advantages. The flap, the thickness of which can be as thin as 2 ~ 3 mm, is especially suitable for soft tissue defects in the hand. Because of the thin character, this flap is more flexible, easy to trim, easy to mold and expand, and suitable to repair three‐dimensional and irregular defects in the hand. The flap size does not need to be much larger than that of the defect. The inset of the flap is easy and does not affect the circulation. As the ALT deep fascia flap is uniformly thin rather than partially or marginally thin, it is more suitable for the coverage of small defects. Multilobed deep fascia flaps can also be harvested according to the need for defects.

Since the ALT deep fascia flap does not include the skin and subcutaneous tissue and the sensory nerve is not destroyed, the donor site can be closed directly without paresthesia. The ALT deep fascia flap with a reliable blood supply easily survives. All cases in this study survived successfully, and there was no vascular crisis. This may be due to the small volume, low demand for blood supply, and high tolerance of deep fascia flaps. Compared with other thinned flaps, the ALT deep fascia flap does not need a microsurgical thinning procedure. Therefore, it is more time‐saving, has less bleeding and has a lower risk.

### Surgical Skills

In the clinical work, we found that most of the reliable perforators of the ALT flap were located in the middle thigh. A small part of them penetrated from the intermuscular septum between the RF and VL, and most of them penetrated from the part near this intermuscular septum line of the VL. Therefore, we propose a new area for preoperative Doppler detection, as mentioned earlier (Fig. 1), that is, the area within the lateral 3 cm of “line a” and within the upper and lower 5 cm of “point A”. Compared with the traditional circular area located at the midpoint of the iliac‐patella line^8^, this area is helpful for detecting perforators more accurately and designing flaps more perfectly. We introduce an operative approach for perforator dissection, with the lateral margin of the flap being the first incision in the operation. Turning the flap inward, the incision is more convenient for the surgeon sitting on the outside of the patient to view the operation area and to dissect the perforators in the VL. When the main pedicle needs to be detected, the upper margin of the incision should be extended toward the beginning of the femoral artery, and then the pedicle can be exposed between the RF and VL.

The bipolar electrocoagulation technique is as follows: When dissecting the perforators in the VL, muscle fibers with a diameter of 3–5 mm are carefully separated by a small hemostat, then a length of 2–3 mm is scorched by bipolar coagulation, and finally, they are cut off at the center of the scab. Repeating this procedure, all the fibers on the surface of perforators can be cut off, and the branches of perforators are ligated at the same time. In our experience, there is no need for perforators to take any fibers on it. As long as it is handled carefully, it will not cause vascular injury or spasm. This idea can reduce the amount of fibers that need to be cut off, simplify the operation, reduce operation time, and reduce bleeding. To avoid muscular herniation^29^, it is only necessary to suture the subcutaneous region and skin. The deep fascia should not be repaired when closing the donor area. If the deep fascia flap is small, the deep fascia gap should be purposely enlarged. After the flap operation, the pulsation of the pedicle artery was detected by Doppler on the skin a little bit away from the arterial anastomosis to observe the circulation of the deep fascia flap. When changing the dressing, observe the color, luster and edge bleeding of the deep fascia flap and then cover it with Vaseline oil gauze to keep moist.

### Limitations of the Study

The recipient area requires two operations, and its sensation is not excellent. In addition, if the deep fascia flap is harvested too large, whether the circulation of the donor site skin would be affected still needs further study. Because of the relatively small patient population and some short follow‐up time, it is difficult to obtain a more definite conclusion. In future work, we will continue to increase the number of cases to present a more convincing conclusion. Based on the similar requirements for reconstruction of the hand and foot, we will extend this method to other shallow regions, such as the forearm, foot and leg.

### Conclusion

This retrospective case series demonstrates that the free ALT deep fascia flap, which can avoid the bulky appearance of the traditional ALT flap and involves fewer complications at the donor site, is suitable for the reconstruction of soft tissue defects in the hand, especially irregular defects in obese and/or female patients. It can provide very good outcomes both functionally and aesthetically.

## Authorship Declaration

All authors listed meet the authorship criteria according to the latest guidelines of the International Committee of Medical Journal Editors, and all authors are in agreement with the manuscript.

## Competing Interests

The authors declare that they have no competing interests.

## Ethics Approval and Consent to Participate

All procedures were part of the standard medical care. As this study has a retrospective design, the need for informed consent was waived by the Institutional Review Board.
